# Supplementary material for: Safety and Efficacy of Crizotinib in Combination with Temozolomide and Radiotherapy in Patients with Newly Diagnosed Glioblastoma: Phase Ib GEINO 1402 Trial
Source: Cancers (Basel). 2022 May 12;14(10):2393. doi: 10.3390/cancers14102393 (PMC9139576; doi:10.3390/cancers14102393)
Supplement: Supplementary file 1 [file cancers-14-02393-s001.zip › cancers-1695332-supplementary.pdf]

cancers

MDPI

Supplementary Materials

Safety and Efficacy of Crizotinib in Combination with Temozolomide and Radiotherapy in Patients with Newly Diagnosed Glioblastoma: Phase Ib GEINO 1402 Trial

María Martínez-García [1,2,3,*], Guillermo Velasco [4,5], Estela Pineda [6], Miguel Gil-Gil [7], Francesc Alameda [8], Jaume Capellades [9], Mari Cruz Martín-Soberón [10], Israel López-Valero [4], Elena Tovar [4], Palmira Foro [11], Álvaro Taus [1,3], Montserrat Arumi [7], Aurelio Hernández-Laín [12] and Juan Manuel Sepúlveda-Sánchez [10,*]

[1] Medical Oncology Department, Hospital del Mar, 08003 Barcelona, Spain; ataus@psmar.cat
[2] Medical Oncology Department, Centro Integral Oncológico Clara Campal HM Delfos, 08023 Barcelona, Spain
[3] Cancer Research Program, Instituto Hospital del Mar de Investigaciones Médicas, 08003 Barcelona, Spain
[4] Biochemistry and Molecular Biology Department, School of Biology, Complutense University, 28040 Madrid, Spain; gvelasco@ucm.es (G.V.); ilvalero@ucm.es (I.L.-V.); elenatov@ucm.es (E.T.)
[5] Instituto de Investigación Sanitaria San Carlos (IdISSC), 28040 Madrid, Spain
[6] Translational Genomics and Targeted Therapeutics in Solid Tumors, August Pi i Sunyer Biomedical Research Institute (IDIBAPS), Medical Oncology Department, Hospital Clínic, 08036 Barcelona, Spain; epineda@clinic.cat
[7] Medical Oncology Department, Institut Català d'Oncologia L'Hospitalet, 08908 L'Hospitalet de Llobregat, Spain; mgilgil@iconcologia.net (M.G.-G.); marumi@psmar.cat (M.A.)
[8] Pathology Department, Hospital del Mar, Universitat Autònoma de Barcelona, 08193 Barcelona, Spain; 86780@parcdesalutmar.cat
[9] Radiology Department, Hospital del Mar, 08003 Barcelona, Spain; jcapellades@parcdesalutmar.cat
[10] Medical Oncology Department, Hospital Universitario 12 de Octubre, 28041 Madrid, Spain; mcms.207@gmail.com
[11] Radiation Oncology Department, Hospital del Mar, 08003 Barcelona, Spain; pforo@parcdesalutmar.cat
[12] Pathology Department (Neuropathology), Hospital 12 de Octubre Research Institute (imas12), 28041 Madrid, Spain; aurelio.hlain@salud.madrid.org
* Correspondence: mariamartinezgarcia@psmar.cat (M.M.-G.); sepulvedasanchez@seom.org (J.M.S.-S.); Tel.: +34-932-4831-37 (M.M.-G.); +34-913-9080-00 (J.M.S.-S.)

Simple Summary: Most patients with glioblastoma, the most frequent primary brain tumor in adults, develop resistance to standard first-line treatment combining temozolomide and radiotherapy. Signaling through the hepatocyte growth factor receptor (c-MET) and the midkine (ALK ligand) promotes gliomagenesis and glioma stem cell maintenance, contributing to the resistance of glioma cells to anticancer therapies. This trial reports for the first time that the addition of crizotinib, an ALK, ROS1, and c-MET inhibitor, to standard RT and TMZ is safe and resulted in a promising efficacy for newly diagnosed patients with glioblastoma.

Abstract: Background**:** MET-signaling and midkine (ALK ligand) promote glioma cell maintenance and resistance against anticancer therapies. ALK and c-MET inhibition with crizotinib have a preclinical therapeutic rationale to be tested in newly diagnosed GBM. Methods**:** Eligible patients received crizotinib with standard radiotherapy (RT)/temozolomide (TMZ) followed by maintenance with crizotinib. The primary objective was to determine the recommended phase 2 dose (RP2D) in a 3 + 3 dose escalation (DE) strategy and safety evaluation in the expansion cohort (EC). Secondary objectives included progression-free (PFS) and overall survival (OS) and exploratory biomarker analysis. Results: The study enrolled 38 patients. The median age was 52 years (33–76), 44% were male, 44% were MGMT methylated, and three patients had IDH1/2 mutation. In DE, DLTs were reported in 1/6 in the second cohort (250 mg/QD), declaring 250 mg/QD of crizotinib as the RP2D for the EC. In the EC, 9/25 patients (32%) presented grade ≥3 adverse events. The median follow up was 18.7 months (m) and the median PFS was 10.7 m (95% CI, 7.7–13.8), with a 6 m PFS and 12 m PFS of 71.5% and 38.8%, respectively. At the time of this analysis, 1 died without progression and

24 had progressed. The median OS was 22.6 m (95% CI, 14.1–31.1) with a 24 m OS of 44.5%. Molecular biomarkers showed no correlation with efficacy. Conclusions: The addition of crizotinib to standard RT and TMZ for newly diagnosed GBM was safe and the efficacy was encouraging, warranting prospective validation in an adequately powered, randomized controlled study.

Keywords: glioblastoma; crizotinib; temozolomide; radiotherapy; midkine

Table S1. Treatment-related Adverse Events classified by study phase, crizotinib dose and grade. * Dose limiting toxicities (DLTs). Data cut-off at 10%. Toxicities here reported were causally related to any investigational medicinal product.

Phase	Dose Escalation Phase			Dose Expansion Phase	TOTAL
Crizotinib dose	200 mg/QD	250 mg/QD	200 mg/BID	250 mg/QD	Any
Grade	Any grade	Any grade	Any grade	Any grade	Any grade
Number of patients N(%)	3 (100%)	6 (100%)	3 (100%)	25 (100%)	37 (100%)
Nausea	2 (66.7)	4 (66.7)	3 (100)	16 (64.0)	25 (67.6)
Fatigue	2 (66.7)	3 (50.0)	2 (66.7)	16 (64.0)	23 (62.2)
Transaminitis	2 (66.7)	3 (50.0)	1 (33.3)	10 (40.0)	16 (43.2)
Neutrophil count decreased	2 (66.7)	3 (50.0)	1 (33.3)	6 (24.0)	12 (32.4)
Platelet count decreased	2 (66.7)	2 (33.3)	0 (0.0)	7 (28.0)	11 (29.7)
Diarrhea	1 (33.3)	2 (33.3)	2 (66.7)	6 (24.0)	11 (29.7)
Anorexia	0 (0.0)	2 (33.3)	1 (33.3)	8 (32.0)	11 (29.7)
Vomiting	0 (0.0)	1 (16.7)	1 (33.3)	8 (32.0)	10 (27.0)
Constipation	1 (33.3)	1 (16.7)	2 (66.7)	5 (20.0)	9 (24.3)
Alopecia	1 (33.3)	3 (50.0)	1 (33.3)	2 (8.0)	7 (18.9)
Lymphocyte count decreased	1 (33.3)	0 (0.0)	0 (0.0)	5 (20.0)	6 (16.2)
Abdominal pain	1 (33.3)	0 (0.0)	1 (33.3)	3 (12.0)	5 (13.5)
White blood cell decreased	0 (0.0)	0 (0.0)	0 (0.0)	4 (16.0)	4 (10.8)
Dyspepsia	1 (33.3)	1 (16.7)	0 (0.0)	2 (8.0)	4 (10.8)
Dysgeusia	0 (0.0)	1 (16.7)	1 (33.3)	2 (8.0)	4 (10.8)
Alanine aminotransferase increased	0 (0.0)	0 (0.0)	0 (0.0)	4 (16.0)	4 (10.8)

Table S2. Treatment-related Adverse Events Grade ≥3 classified by treatment phase. Toxicities here reported were causally related to any investigational medicinal product.

**Event**	**Concomitant Treatment**		**Adjuvant Treatment**		**Maintenance Treatment**	
	**Dose Escalation**	**Dose Expansion**	**Dose Escalation**	**Dose Expansion**	**Dose Escalation**	**Dose Expansion**
	**N (%)**	**N (%)**	**N (%)**	**N (%)**	**N (%)**	**N (%)**
	**N= 12**	**N= 25**	**N= 10**	**N= 24**	**N= 7**	**N= 13**
Transaminitis	2 (16.7%)	6 (24%)	-	-	-	-
Neutropenia	3 (25%%)	-	2 (20%)	1 (4.3%)	-	-
Thrombocytopenia	-	1 (4%)	2 (20%)	1 (4.3%)	-	-
Lymphopenia	1 (8.3%)	1 (4%)	-	1 (4.3%)	-	-
Cholecystitis	-	-	-	1 (4.3%)	-	-
Hepatic toxicity	-	-	-	1 (4.3%)	-	-
Edema	-	-	-	-	-	1 (7.7%)
Constipation	1 (8.3%)	-	-	-	-	-
Hypophosphatemia	1 (8.3%)	-	-	-	-	-
Fatigue	-	-	1 (10%)	-	-	-
Total of events	16		10		1	

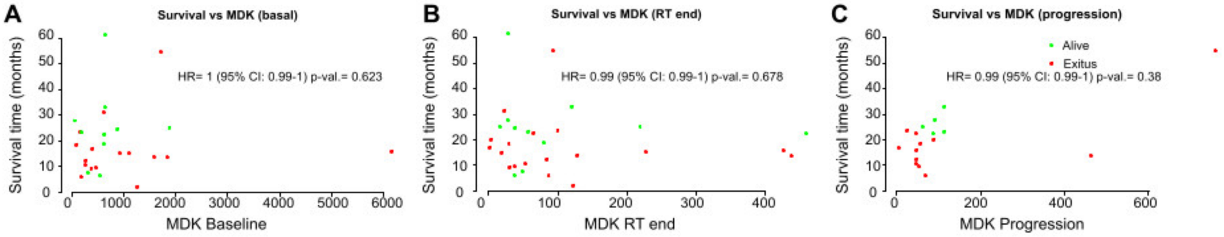

Figure S1. Correlation of survival and MDK levels at baseline (**A**), at the end of radiotherapy (**B**), and at the end of treatment (**C**). Scatter plot representing the two-dimensional space the patients according to their survival (Y axis) and MD levels (X axis). Those patients that died are represented as red dots, and those that were alive or censored for survival as green dots. Cox model Hazard ratios and p values are depicted within each graph.
